# Selective Complement Inhibition in Anti-p200 Pemphigoid: Immune Infiltrate Profiles and Therapeutic Implications Compared to Bullous Pemphigoid

**DOI:** 10.3390/biom16020182

**Published:** 2026-01-23

**Authors:** Shirin Emtenani, Tina Rastegar Lari, Charlotte Kiehne, Nina van Beek, Maike M. Holtsche, Enno Schmidt

**Affiliations:** 1 Lübeck Institute of Experimental Dermatology, University of Lübeck, 23562 Lübeck, Germany; 2 Department of Dermatology, Schleswig-Holstein University Hospital, Campus Lübeck, 23562 Lübeck, Germany; 3 Department of Dermatology, University of Oldenburg, 26129 Oldenburg, Germany

**Keywords:** autoimmune blistering disease, anti-p200 pemphigoid, bullous pemphigoid, neutrophil, eosinophil, complement fixation, complement inhibition

## Abstract

Anti-p200 pemphigoid is an autoimmune blistering disease (AIBD) caused by autoantibodies against laminin β4 and/or γ1, and clinically resembles bullous pemphigoid (BP) as well as the inflammatory variant of epidermolysis bullosa acquisita (EBA). All three diseases show IgG and/or C3 deposition along the cutaneous basement membrane zone (BMZ). Although complement activation is central to BP and EBA pathogenesis, its role in anti-p200 pemphigoid remains unclear. To investigate this, we analyzed inflammatory infiltrates in lesional and perilesional skin from anti-p200 pemphigoid patients (*n* = 11), revealing a neutrophil-predominant pattern, with mixed neutrophil–eosinophil infiltrates in 81% of cases, which contrasted with the eosinophil-rich infiltrates typical of BP. Infiltrating neutrophils expressed C5aR1 and C5aR2. Complement fixation test (CFT) of patient sera demonstrated C3c deposition at the BMZ in 40% (20/50) of anti-p200 pemphigoid cases and 87% (13/15) of BP cases. Patients in both cohorts could be stratified into high, mild, and non-complement-fixating groups. Pharmacological inhibition of C1s (sutimlimab), C3 (compstatin), C5 (tesidolumab), or C5aR1 (avacopan) significantly blocked C3c or C5 deposition in vitro. These findings indicate that selective blockade of the classical, alternative, or terminal complement pathways effectively prevents BMZ complement deposition, highlighting pathway-specific complement inhibition as a potential therapeutic strategy for anti-p200 pemphigoid.

## 1. Introduction

Autoimmune blistering diseases (AIBDs) are rare, chronic disorders characterized by autoantibodies against structural components of the skin and/or mucous membranes, leading to blister formation. Pemphigoid diseases are subepidermal AIBDs that include bullous pemphigoid (BP), mucous membrane pemphigoid, pemphigoid gestationis, linear IgA disease, epidermolysis bullosa acquisita (EBA), and anti-p200 pemphigoid [1,2,3,4]. Pathogenesis involves autoantibody binding to basement membrane zone (BMZ) proteins, complement activation, recruitment of inflammatory cells, and release of reactive oxygen species (ROS) and proteolytic enzymes, culminating in epidermal–dermal separation and subepidermal blistering [5].

Anti-p200 pemphigoid is a rare pemphigoid disease driven by autoantibodies against laminin β4 and/or γ1 [6,7,8,9]. Diagnosis relies on direct immunofluorescence (IF) with linear IgG and/or C3 deposition at the BMZ in perilesional skin and dermal IgG binding along the floor of the artificial blister by indirect IF on human salt-split skin. Diagnosis requires detection of serum reactivity with the 200 kDa p200 protein in human skin extracts by immunoblotting, and/or with recombinant laminin γ1 and/or laminin β4 [6,10]. For serum reactivity against laminin β4, a standardized, highly specific, and sensitive indirect IF-based assay is widely available [11]. Clinically, patients present with tense blisters, erosions, and urticarial plaques on the trunk and extremities, resembling BP and the inflammatory variant of EBA [6,12]. Mucosal involvement, most frequently affecting oral, anogenital, or conjunctival mucosa, occurs in approximately 40% of patients, which is higher than in BP and EBA [12,13]. Histopathologically, anti-p200 pemphigoid typically presents with subepidermal blisters containing predominantly neutrophilic or mixed neutrophilic–eosinophilic infiltrates [6,12].

BP, the most prevalent AIBD, is characterized by autoantibodies directed against the hemidesmosomal proteins BP180 and BP230 [2,4]. Complement deposition, particularly C3, is a hallmark of both BP and anti-p200 pemphigoid, suggesting a role for complement activation in disease pathogenesis [2,6,14]. Diagnostic complement markers such as C4d, well-established in BP, have recently been investigated in anti-p200 pemphigoid [15,16,17]. Nevertheless, the substantial clinical and histopathological overlap between anti-p200 pemphigoid and BP/EBA complicates differential diagnosis and therapeutic decision-making.

Given the serious side effects of corticosteroids, complement-targeted interventions represent a promising therapeutic strategy. However, the precise contribution of complement activation to tissue damage in anti-p200 pemphigoid remains poorly defined. In this study, we characterized the histopathological differences between anti-p200 pemphigoid and BP, and investigated the role of complement activation and its pharmacological modulation in vitro, thereby informing targeted therapeutic approaches.

## 2. Materials and Methods

### 2.1. Ethics Approval

Skin biopsies (formalin-fixed, paraffin-embedded; FFPE, *n* = 11) from newly diagnosed anti-p200 pemphigoid patients, along with sera from newly diagnosed patients with anti-p200 pemphigoid (*n* = 50) and BP (*n* = 15), were obtained from the Department of Dermatology, Schleswig-Holstein University Hospital (UKSH), Lübeck. In brief, anti-p200 pemphigoid was characterized by (i) tense blisters on normal and erythematous skin; (ii) linear deposits of IgG and⁄or C3 at the dermal–epidermal junction (DEJ) detected by direct IF microscopy; (iii) binding of IgG deposition along the dermal side of the split by indirect IF microscopy on 1 mol L^−1^ NaCl-split normal human skin; (iv) reactivity of serum IgG antibodies with a 200-kDa protein by Western blotting using extracts generated from normal human dermis, and (v) ELISA employing a recombinant monomeric C-terminal fragment of human laminin c1 (hLAMC1-cterm) [18]. Skin specimens used for the complement fixation test (CFT) were collected from patients undergoing breast or abdominal reduction surgery after written informed consent had been obtained. The study was approved by the Ethics Committee of the University of Lübeck (2022-469, approved on 13 March 2023; 2023-326, approved on 4 April 2023) and conducted in accordance with the Declaration of Helsinki.

### 2.2. Immunohistochemistry (IHC)

FFPE tissue samples from anti-p200 pemphigoid patients (*n* = 11) were processed using the BenchMark Ultra autostainer (Roche, Basel, Switzerland). Sections (4 µm thick) were deparaffinized, rehydrated, and pretreated with the OptiView DAB IHC Detection Kit (Roche) and stained with the following antibodies: rabbit monoclonal anti-human CD3 antibody (Clone 2GV6; ready-to-use, 16 min incubation at room temperature (RT); Roche), rabbit monoclonal anti-human CD4 (Clone SP35, ready-to-use, 16 min incubation; Roche), rabbit monoclonal anti-human CD8 (Clone SP57; ready-to-use, 16 min incubation; Roche), rabbit monoclonal anti-human CD20 (Clone L26; ready-to-use, 16 min incubation; Roche), mouse monoclonal anti-human CD30 (Clone Ber-H2; ready-to-use, 32 min incubation; Roche), mouse monoclonal anti-CD68 (Clone KP-1; ready-to-use, 12 min incubation; Roche), rabbit monoclonal anti-human CD56 (Clone MRQ-42; ready-to-use, 16 min incubation; Roche), mouse anti-human CD138 monoclonal (Clone B-A38; ready-to-use, 20 min incubation; Cell Marque, Rocklin, CA, USA), and rabbit polyclonal anti-human myeloperoxidase (MPO; ready-to-use, 16 min incubation; Roche) antibodies. Eosinophils were detected by routine hematoxylin and eosin (H&E) staining.

To analyze the cellular sources of C5aR1 and C5aR2 expression, double IF staining was performed on 6 µm FFPE sections from 11 anti-p200 pemphigoid patients, as previously described [19]. After deparaffinization and graded ethanol dehydration, heat-induced antigen retrieval in citrate buffer (pH 6.0) was conducted for 10 min in a pressure cooker. Slides were washed in PBS with 0.05% Tween-20 and blocked with 5% (*v*/*v*) normal donkey serum (Jackson ImmunoResearch Laboratories, Suffolk, UK) for 1 h at RT. Sections were incubated overnight at 4 °C with rabbit anti-human C5aR1 or C5aR2 antibodies (ThermoFisher Scientific, Dreieich, Germany) in combination with mouse anti-human myeloperoxidase (MPO; clone 392105; R&D Systems, Minneapolis, MN, USA) or eosinophil peroxidase (EPX; clone MM25-82.2; Mayo Clinic, Scottsdale, AZ, USA) antibodies. As secondary antibodies, Alexa Fluor 594-AffiniPure donkey anti-rabbit IgG (Jackson ImmunoResearch Laboratories) and Alexa Fluor 488 goat anti-mouse IgG (ThermoFisher Scientific) were applied for 1 h at RT. Slides were mounted with DAPI Fluoromount G^®^ (Southern Biotech, Birmingham, AL, USA). Images were captured using a Keyence BZ-X810 microscope (Keyence GmbH, Neu-Isenburg, Germany). For quantification of inflammatory infiltrates, cells were manually counted in two representative fields per lesional and perilesional sections for each patient, ideally selecting one from each side of the section. Counting was performed at 40× magnification for IHC or 10× magnification for double IF staining, with all analyses conducted in a blinded manner.

### 2.3. Complement Fixation Test (CFT)

CFT was performed as previously described, with slight modifications [20,21]. Human skin cryosections from healthy donors were incubated with heat-inactivated sera (diluted 1:10 in PBS) from BP (*n* = 15) or anti-p200 pemphigoid (*n* = 50) patients overnight at RT in a dark, humid chamber. Following washing, fresh pooled hirudin plasma from five healthy donors (diluted 1:5 in veronal buffered saline; Sigma-Aldrich, St. Louis, MO, USA) was added and incubated for 1.5 h at 37 °C. Sections were then stained with FITC-conjugated rabbit anti-human C3c (Dako, Carpinteria, CA, USA) or FITC-conjugated human complement C5 AssayLite^®^ (AssayPro LLC, St. Charles, MO, USA) antibodies for 1 h at RT. Nuclei were counterstained with DAPI Fluoromount-G^®^. Images were acquired using a Keyence microscope. Complement deposition intensity was scored semi-quantitatively in a blinded fashion as strong (2), mild (1), or absent (0).

### 2.4. In Vitro Complement Inhibition

Sera with strong complement-fixating activity (*n* = 3) were pooled for pharmacological testing. Normal human serum (NHS) served as a negative control. The CFT was performed in the presence or absence of selective inhibitors targeting specific complement components (Selleckchem, Houston, TX, USA): C1s (sutimlimab, 10–100 μg/mL), C3 (compstatin, 5–50 μM), C5 (tesidolumab, 10–100 μg/mL), and C5aR1 (avacopan, 5–50 μM). Tinzaparin sodium (1–100 IU; LEO Pharma, Ballerup, Denmark) and 1 mM EDTA were used as positive inhibitory controls [20]. Slides were examined by microscopy, and complement deposition was analyzed in a blinded manner.

### 2.5. Statistical Analysis

Data were analyzed using GraphPad Prism (version 10.0; GraphPad Software, San Diego, CA, USA). Significance was determined by one-way ANOVA followed by Dunnett’s post hoc multiple-comparisons test. Results are expressed as mean ± standard error of the mean (SEM). Statistical significance was defined as *p* < 0.05.

## 3. Results

### 3.1. Neutrophils Predominate Among Inflammatory Infiltrates in Anti-p200 Pemphigoid Skin

**Figure 1:**
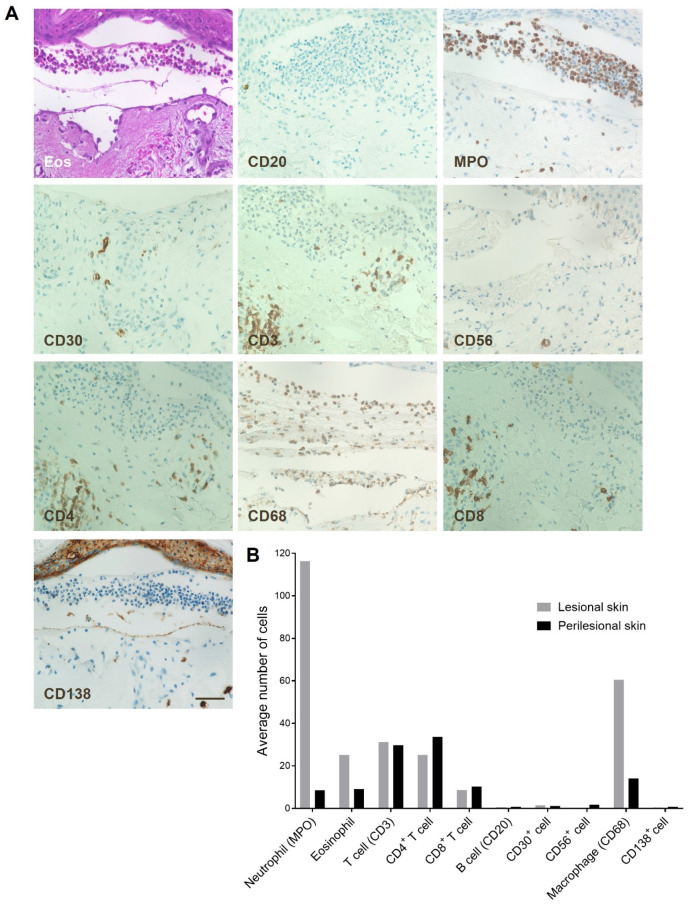
Immunohistochemical (IHC) characterization of cell infiltrates in anti-p200 pemphigoid skin. (**A**) Representative IHC images of lesional skin biopsies from anti-p200 pemphigoid patients (*n* = 11), demonstrating prominent dermal infiltration by distinct immune cell populations. Staining was performed for eosinophils (H&E), B cells (CD20), neutrophils (myeloperoxidase; MPO), activated lymphocytes (CD30), T cells (CD3, CD4, and CD8), natural killer cells (CD56), macrophages (CD68), and plasma cells (CD138). (**B**) Quantitative analysis of immune cell populations in perilesional versus lesional skin. Neutrophils were the most abundant inflammatory cell type in both compartments, followed by macrophages, T cells, and eosinophils. Other subsets were detected at low frequencies. Scale bar: 50 μm.

IHC analysis of FFPE lesional and perilesional skin biopsies from anti-p200 pemphigoid patients identified neutrophils as the predominant immune cell population, comprising approximately 43% and 9% in lesional and perilesional skin, respectively, followed by CD68^+^ macrophages (22% and 13%, respectively), CD3^+^ T cells (12% and 27%, respectively), and eosinophils (9% and 8%, respectively; Figure 1). Other immune cell subsets, including B cells (CD20^+^), natural killer cells (CD56^+^), activated lymphocytes (CD30^+^), and CD138^+^ (plasma cells), were detected only infrequently (Figure 1). Notably, the average numbers of neutrophils and eosinophils were markedly higher in lesional skin than in perilesional skin. A mixed neutrophil–eosinophil infiltrate was observed in 9 of 11 (81%) anti-p200 pemphigoid patients.

### 3.2. C5a Receptors Are Expressed by Neutrophils in Anti-p200 Pemphigoid Skin

**Figure 2:**
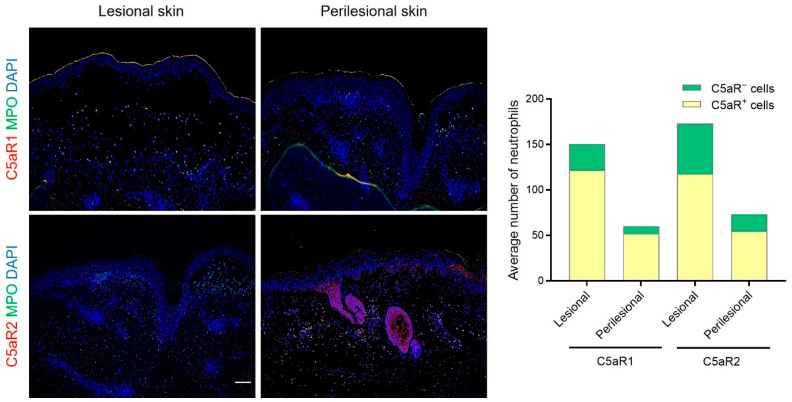
C5a receptor expression in neutrophils in anti-p200 pemphigoid skin. Quantitative analysis of C5aR1^+^ and C5aR2^+^ cells in perilesional and lesional skin biopsies from patients with anti-p200 pemphigoid (*n* = 11). Double IF staining shows colocalization of C5aR1 or C5aR2 with neutrophils (myeloperoxidase (MPO); green). Colocalized cells (C5aR^+^/MPO^+^) appear yellow in merged images. Nuclei were counterstained with DAPI (blue). Scale bar, 100 μm.

To explore the contribution of complement activation in anti-p200 pemphigoid, we performed double IF staining to assess C5aR1 and C5aR2 expression on neutrophils, the most abundant cell type within the inflammatory infiltrate of anti-p200 pemphigoid skin. The vast majority of neutrophils expressed C5aR1 and/or C5aR2. The average total number of C5aR1^+^ and/or C5aR2^+^ cells was higher in anti-p200 pemphigoid lesions compared with perilesional skin. In contrast, the percentage of C5aR1^+^ and/or C5aR2^+^ neutrophils was slightly lower in lesional skin (80% and 68%, respectively) than in perilesional skin (86% and 74%, respectively) (Figure 2).

### 3.3. Sera from Anti-p200 Pemphigoid and BP Patients Fix Complement at the Cutaneous BMZ

**Figure 3:**
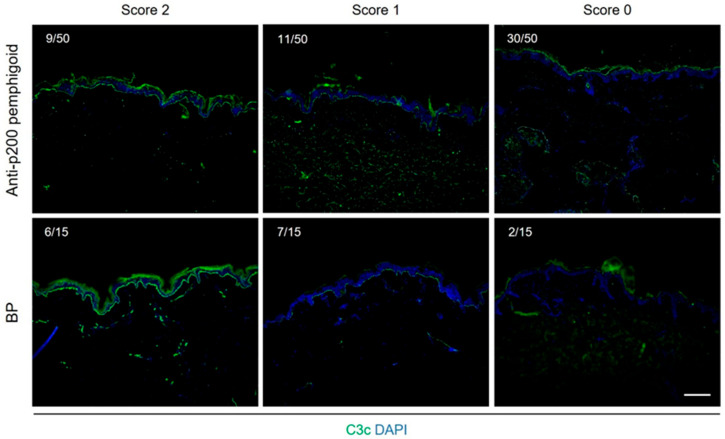
Serum autoantibodies from anti-p200 pemphigoid and BP patients induce complement deposition at the basement membrane zone (BMZ). Complement fixation test (CFT) was performed to assess the complement-fixing capacity of autoantibodies in sera from patients with anti-p200 pemphigoid (*n* = 50) and BP (*n* = 15). Autoantibodies bound to the BMZ of normal human skin cryosections were incubated with fresh pooled human complement (hirudin plasma from five healthy donors), followed by detection of deposited C3c using FITC-conjugated anti-human C3c antibody. Complement deposition (green) was evaluated semi-quantitatively: strong (score 2), mild (score 1), or absent (score 0). Representative images illustrate linear C3c deposition along the BMZ in CFT-positive samples (scores 2 and 1). Nuclei were counterstained with DAPI (blue). Scale bar, 100 μm.

Sera from 50 anti-p200 pemphigoid and 15 BP patients were examined using CFT. Based on C3c deposition, sera were categorized as high, mild, or non-complement fixating. In the anti-p200 pemphigoid cohort, 20 sera (40%) were CFT-positive: 9 showed strong C3c deposition (score 2), and 11 showed mild deposition (score 1). The remaining 30 sera (60%) exhibited no complement-fixing activity. In contrast, 13 of 15 BP sera (87%) induced C3c deposition at the BMZ (6 strong, 7 mild) (Figure 3).

### 3.4. Selective Complement Inhibitors Effectively Suppress Complement Deposition

**Figure 4:**
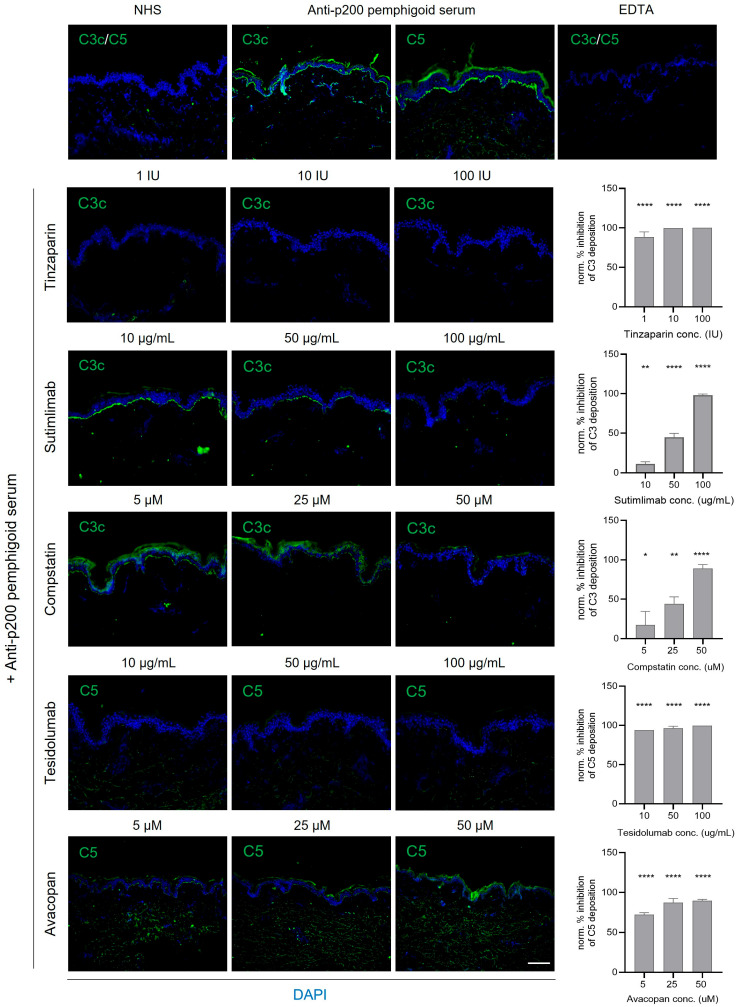
Inhibition of complement fixation by selective inhibitors in an in vitro model of anti-p200 pemphigoid. Human skin cryosections were incubated with heat-inactivated, pooled sera from three anti-p200 pemphigoid patients exhibiting strong C3c/C5-fixating activity, followed by a complement source. Increasing concentrations of selective inhibitors targeting C1s (sutimlimab), C3 (compstatin), C5 (tesidolumab), or C5aR1 (avacopan) were added to the complement source. EDTA and tinzaparin sodium served as positive controls for complement inhibition. BMZ complement fixation (C3c or C5; green) was assessed by IF microscopy and semi-quantified relative to untreated controls (no inhibitor). Sutimlimab and compstatin dose-dependently suppressed C3c deposition, while tesidolumab and avacopan led to almost complete inhibition of C5 complement fixation at all tested concentrations. In contrast, no complement fixation was observed in the presence of normal human serum (NHS). Nuclei were visualized by DAPI (blue) counterstaining. Semi-quantitative analyses were performed using three independent experiments. **** *p* < 0.0001; ** *p* < 0.01; * *p* < 0.05. Scale bar, 100 μm.

**Figure 5:**
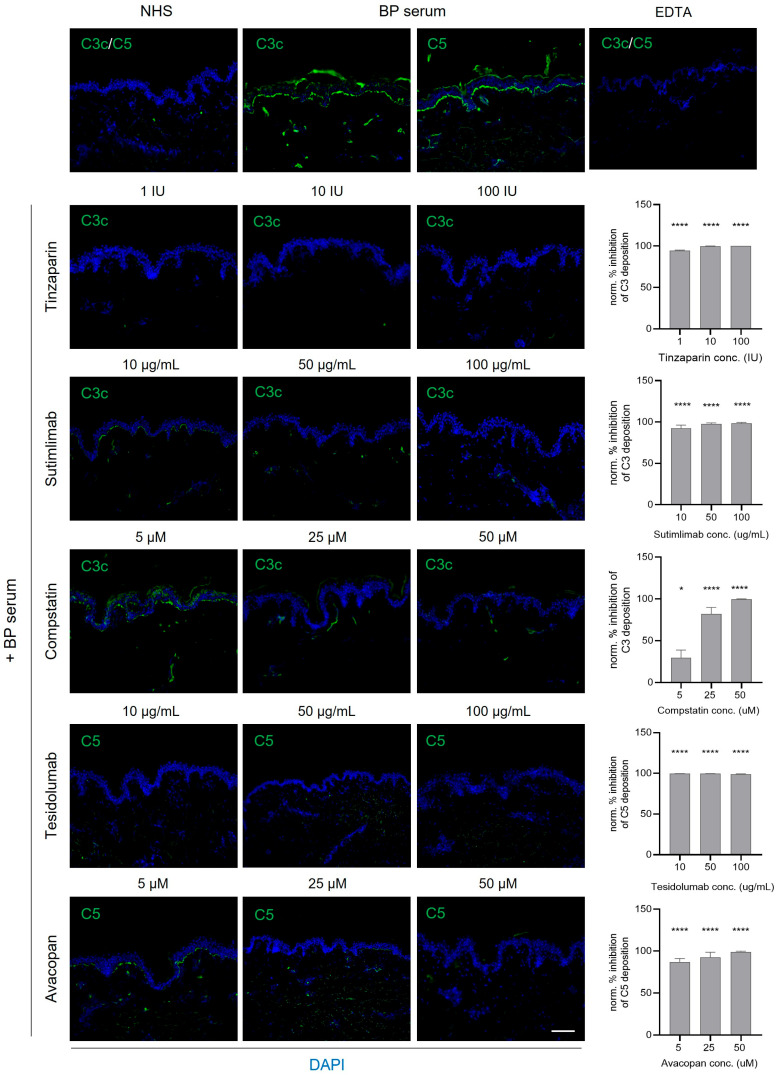
Targeted inhibition of complement deposition induced by BP autoantibodies in an in vitro model. Human skin cryosections were incubated with pooled, heat-inactivated sera from three BP patients with strong C3c/C5 fixation (green). Selective complement inhibitors, including anti-C1s antibody (sutimlimab), C3 inhibitor (compstatin), anti-C5 antibody (tesidolumab), or C5aR1 inhibitor (avacopan), as well as EDTA and tinzaparin sodium (positive controls for complete complement inhibition) were added alongside hirudin plasma as a complement source. Tissues were stained for C3c or C5 deposition, and percentage inhibition of complement deposition was semi-quantified relative to untreated samples. A dose-dependent inhibition of C3c deposition was observed with compstatin, while all other compounds led to a nearly complete C3c or C5 inhibition along the BMZ at all tested concentrations. Normal human serum (NHS) did not induce complement fixation. Nuclei were visualized by DAPI (blue) counterstaining. Data from three independent experiments were used for semi-quantitative analysis. **** *p* < 0.0001; * *p* < 0.05. Scale bar: 100 μm.

We next evaluated the effects of selective complement inhibitors on in vitro complement activation induced by patient autoantibodies using CFT. The C1s inhibitor sutimlimab and the C3 inhibitor compstatin remarkedly blocked linear C3c deposition along the BMZ (Figure 4 and Figure 5). Likewise, the C5 inhibitor tesidolumab and the C5aR1 inhibitor avacopan significantly reduced BMZ C5 deposition. Tinzaparin sodium served as a positive inhibitory control [20]. Collectively, all four agents effectively suppressed solid-phase complement activation induced by serum autoantibodies from patients with anti-p200 pemphigoid (Figure 4) or BP (Figure 5) in vitro.

## 4. Discussion

The histopathological and clinical features of anti-p200 pemphigoid frequently resemble those of BP [12,22]. In this study, we characterized the composition of the inflammatory infiltrate in both lesional and perilesional skin of anti-p200 pemphigoid patients using IHC. Both lesional and perilesional skin biopsies revealed predominantly neutrophilic infiltrates within the superficial dermis, whereas eosinophils were less prominent. In contrast, BP skin typically exhibits an upper dermal infiltrate rich in lymphocytes and eosinophils, with neutrophils observed only occasionally [4,23]. Consistent with this, in a cohort of 136 patients with BP, eosinophilic infiltrate was observed in the vast majority of cases (*n* = 122; 89.7%), whereas neutrophilic infiltrates were less frequently encountered (*n* = 22; 16.2%) [23]. These histological differences may assist in differential diagnosis; however, histology alone is insufficient and should be interpreted together with serological analyses or DIF testing [24]. Furthermore, lesional anti-p200 pemphigoid skin contained higher absolute numbers of C5aR1^+^ and/or C5aR2^+^ cells, whereas the reduced proportion of receptor-positive neutrophils likely reflects a broader inflammatory infiltrate in established lesions. In contrast, perilesional BP skin showed a slightly higher proportion of C5aR1^+^ than C5aR2^+^ neutrophils [19].

Complement deposition in perilesional skin, as detected by direct IF, and in FFPE lesional tissue by immunoperoxidase staining in BP patients suggests that complement activation contributes to lesion formation. This concept is further supported by experimental mouse models of BP, in which complement activation is an essential component of lesion formation [25,26,27]. However, the pathogenic role of complement in anti-p200 pemphigoid remains unexplored in vivo due to the lack of laminin β4 expression in rodent skin and the failure to induce skin lesions in mice by injection of anti-laminin γ1 IgG or immunization with recombinant forms of this protein [28,29]. In rodents, the development of humanized skin graft models may represent an option to validate complement dependency in vivo. Accordingly, evidence regarding complement activation in human anti-p200 pemphigoid remains limited. Previous studies reported C3c deposition in approximately 78% of anti-p200 pemphigoid skin biopsies compared to 85% of BP skin biopsies [12,30]. Similarly, C4d deposition at the cutaneous BMZ has been observed in 60% of lesional skin biopsies from anti-p200 pemphigoid patients, compared to 70% in BP lesions [15,17].

We assessed the complement-fixating potential of anti-p200 pemphigoid IgG using the CFT and examined pharmacological blockade of autoantibody-induced complement activation in vitro. CFT is a well-established assay long applied to the study of BP and pemphigoid gestationis [31]. In this assay, complement-inactivated patient sera were incubated with healthy human skin cryosections. Normal human hirudin plasma was then added as a complement source, with or without specific inhibitors. Among the 50 anti-p200 pemphigoid sera, 20 (40%) induced BMZ complement fixation, compared with 13 of 15 (87%) BP sera, indicating a stronger C3-fixating capacity of BP autoantibodies. This difference is consistent with trends observed in DIF studies, in which C3 deposition is reported slightly more frequently in BP than in anti-p200 pemphigoid [12,23,30]. Both cohorts could be classified as strongly, mildly, or non-complement-fixing subgroups. This heterogeneity, more pronounced in anti-p200 pemphigoid, supports a pathogenic role for complement while highlighting interindividual variability in effector mechanisms. It also suggests that CFT could potentially identify complement-dependent subsets suitable for targeted therapy. Since sera were collected at initial diagnosis during active disease before systemic immunosuppression, the observed variability is unlikely to be attributable to treatment effects or disease chronicity. Whether complement-fixing capacity correlates with disease severity, mucosal involvement, or epitope specificity (e.g., laminin β4 vs. laminin γ1) remains unknown and warrants investigation in future longitudinal studies.

Selective complement inhibitors efficiently modulated BMZ C3c and C5 deposition in vitro. Inhibition of the classical pathway with the C1s inhibitor sutimlimab or broad inhibition of C3 activation with compstatin prevented autoantibody-induced C3c deposition. Similarly, inhibition at downstream levels using the C5 inhibitor tesidolumab or the C5aR1 inhibitor avacopan significantly reduced C5 deposition. These findings suggest that overlapping complement pathways may be therapeutically targeted in both anti-p200 pemphigoid and BP patients. Low-molecular-weight heparin tinzaparin sodium served as a consistent positive control [20], whereas glucocorticoids do not prevent complement deposition in these in vitro models [32]. These observations are in line with a recent study reporting that both clinical and preclinical complement inhibitors, including anti-C3, anti-factor B, and anti-C5 (eculizumab) monoclonal antibodies, blocked linear BMZ C3c or C5b-9 deposition in CFT [32]. Likewise, a recombinant CD55/CD46 fusion protein inhibited antibody-mediated C3b deposition in vitro, highlighting its potential as a complement-targeted therapy for BP [33]. Moreover, C1s inhibition with the mouse anti-C1s monoclonal antibody TNT003 reduced C4a and C5a generation in human skin cryosections incubated with BP patient serum [34].

This study has several limitations. The relatively small sample size may over- or underestimate certain effects, partly reflecting the rarity of AIBDs. Additionally, the use of supraphysiological inhibitor concentrations may not fully reflect clinical dosing. Nevertheless, these conditions were balanced by the use of high-titer pooled patient sera (score 2 in CFT), ensuring robust BMZ autoantibody binding. Clinically, pemphigoid patients exhibit lower circulating autoantibody titers than those used for the CFT; thus, lower, physiologically relevant inhibitor doses may also be effective. Future studies should correlate CFT results with serum autoantibody levels, disease severity scores (e.g., Bullous Pemphigoid Disease Area Index (BPDAI)), and systemic complement activation markers (e.g., plasma C3a/C5a). Additionally, alternative complement-targeted agents could be evaluated for comparable or superior efficacy at lower concentrations. Assessing systemic complement activation in pemphigoid patients’ sera will also be important to contextualize local findings from skin-based studies.

Currently, corticosteroids are the mainstay of therapy for BP but are associated with numerous, sometimes severe, adverse effects. When resistance to corticosteroids develops, patients exhibit high relapse rates, and disease management becomes challenging [4]. Given the well-known adverse effects of long-term glucocorticoids and immunosuppressants, there remains an unmet need for safe, effective treatments. Sutimlimab (BIVV009), a humanized IgG4 monoclonal antibody targeting C1s, has shown clinical efficacy and regulatory approval in cold agglutinin disease [35], and a phase I trial in BP demonstrated partial or complete abrogation of C3 deposition along the DEJ [36]. Similarly, pegcetacoplan, a PEGylated compstatin derivative, is approved for paroxysmal nocturnal hemoglobinuria (PNH) in patients with inadequate responses to anti-C5 therapy [37,38]. The anti-C5 IgG1 tesidolumab (LFG316) demonstrated promising efficacy in a phase II multicenter trial in PNH patients [39]. Furthermore, nomacopan (coversin), a dual C5 and leukotriene B4 inhibitor, reduced clinical disease activity in BP patients without serious adverse effects in a phase IIa trial [40]. In contrast, a small randomized controlled trial with the anti-C5aR1 inhibitor avdoralimab did not show additional benefit of avdoralimab compared with topical corticosteroids alone [41].

Taken together, these findings highlight the potential of complement-fixation profiling, alongside routine diagnostic tools, to stratify anti-p200 pemphigoid patients and guide future diagnostic and complement-targeted therapeutic studies.

## 5. Conclusions

In summary, our findings support inhibition of ongoing complement fixation and C3a/C5a liberation using selective inhibitors, either as monotherapy or in combination with other immunosuppressive therapies, as a rational, pathway-directed approach to complement-driven AIBDs. The differing complement-fixating capacities of autoantibodies in anti-p200 pemphigoid compared with BP suggest disease-specific complement activation signatures. While the average complement-fixating capacities differ between the two diseases, potentially reflecting distinct therapeutic thresholds, subsets of patients in both cohorts with moderate to strong complement-fixating capacities could benefit from complement-targeted approaches. Future in vivo and clinical studies are warranted to establish optimal dosing and confirm the efficacy of complement blockade in these diseases.

## Data Availability

The original contributions presented in this study are included in the article. Further inquiries can be directed to the corresponding author, S.E., on reasonable request.

## Abbreviations

The following abbreviations are used in this manuscript:

ANCA	antineutrophil cytoplasmic antibody
AIBD	autoimmune blistering disease
BMZ	basement membrane zone
BP	bullous pemphigoid
BPDAI	Bullous Pemphigoid Disease Area Index
CFT	complement fixation test
DEJ	dermal–epidermal junction
DIF	epidermolysis bullosa acquisita
FFPE	formalin-fixed, paraffin-embedded
H&E	hematoxylin and eosin
IF	immunofluorescence
IHC	immunohistochemistry
NHS	normal human serum
PNH	paroxysmal nocturnal hemoglobinuria
ROS	reactive oxygen species
RT	room temperature
SEM	standard error of the mean
